# Bis(μ-2-methyl­quinolin-8-olato)-κ^3^ *N*,*O*:*O*;κ^3^ *O*:*N*,*O*-bis­[(acetato-κ*O*)(methanol-κ*O*)zinc(II)]

**DOI:** 10.1107/S1600536809014214

**Published:** 2009-04-22

**Authors:** Elham Sattarzadeh, Gholamhossein Mohammadnezhad, Mostafa M. Amini, Seik Weng Ng

**Affiliations:** a Department of Chemistry, General Campus, Shahid Beheshti University, Tehran 1983963113, Iran; b Department of Chemistry, University of Malaya, 50603 Kuala Lumpur, Malaysia

## Abstract

The reaction of zinc acetate and 2-methyl-8-hydroxy­quinoline in methanol yielded the centrosymmetric dinuclear title compound, [Zn_2_(C_10_H_8_NO)_2_(CH_3_CO_2_)_2_(CH_3_OH)_2_], which has the Zn atom within a distorted NO_4_ trigonal–bipyramidal coordination geometry. Methanol–acetate O—H⋯O hydrogen bonds link the dinculear units into a linear supra­molecular chain extending parallel to [100].

## Related literature

Unlike 8-hydroxy­quinoline, which yields a large number of metal derivatives, 2-methyl-8-hydroxy­quinoline forms only a small number of metal chelates. Besides a related chloride salt (Sattarzadeh *et al.*, 2009 ▶), there is only one crystal structure report of another zinc derivative; for aqua­bis(2-methyl­quinolin-8-ato)zinc, see: da Silva *et al.* (2007 ▶).
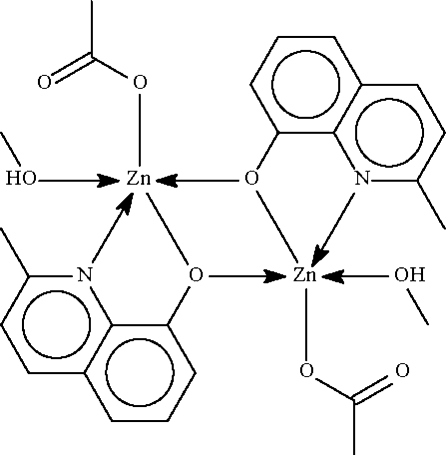


## Experimental

### 

#### Crystal data

[Zn_2_(C_10_H_8_NO)_2_(C_2_H_3_O_2_)_2_(CH_4_O)_2_]
*M* *_r_* = 629.26
Triclinic, 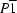

*a* = 6.9496 (1) Å
*b* = 9.6262 (2) Å
*c* = 9.8232 (2) Å
α = 75.241 (1)°
β = 89.688 (1)°
γ = 86.596 (1)°
*V* = 634.32 (2) Å^3^
*Z* = 1
Mo *K*α radiation
μ = 1.95 mm^−1^
*T* = 100 K
0.38 × 0.28 × 0.18 mm

#### Data collection

Bruker SMART APEX diffractometer
Absorption correction: multi-scan (*SADABS*; Sheldrick, 1996 ▶) *T*_min_ = 0.525, *T*_max_ = 0.721
5601 measured reflections
2855 independent reflections
2534 reflections with *I* > 2σ(*I*)
*R*_int_ = 0.042

#### Refinement

*R*[*F* ^2^ > 2σ(*F* ^2^)] = 0.076
*wR*(*F* ^2^) = 0.230
*S* = 1.13
2855 reflections
175 parameters
H-atom parameters constrained
Δρ_max_ = 3.72 e Å^−3^
Δρ_min_ = −1.85 e Å^−3^

### 

Data collection: *APEX2* (Bruker, 2008 ▶); cell refinement: *SAINT* (Bruker, 2008 ▶); data reduction: *SAINT*; program(s) used to solve structure: *SHELXS97* (Sheldrick, 2008 ▶); program(s) used to refine structure: *SHELXL97* (Sheldrick, 2008 ▶); molecular graphics: *X-SEED* (Barbour, 2001 ▶); software used to prepare material for publication: *publCIF* (Westrip, 2009 ▶).

## Supplementary Material

Crystal structure: contains datablocks global, I. DOI: 10.1107/S1600536809014214/tk2424sup1.cif

Structure factors: contains datablocks I. DOI: 10.1107/S1600536809014214/tk2424Isup2.hkl

Additional supplementary materials:  crystallographic information; 3D view; checkCIF report

## Figures and Tables

**Table 1:** Hydrogen-bond geometry (Å, °)

*D*—H⋯*A*	*D*—H	H⋯*A*	*D*⋯*A*	*D*—H⋯*A*
O4—H4⋯O3^i^	0.84	1.88	2.602 (6)	143

Symmetry code: (i) 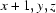.

**Fig. 1.**
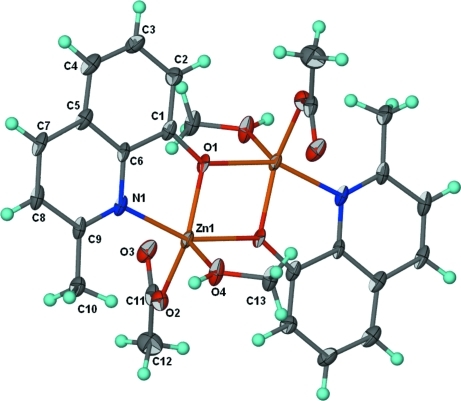
Thermal ellipsoid plot (Barbour, 2001) of dinuclear [Zn(C10H8NO)(CH3OH)(CH3CO2)]2; ellipsoids are drawn at the 70% probability level and H atoms of arbitrary radius. The unlabelled atoms are related by a centre of inversion.

## supplementary crystallographic information

### Experimental

Zinc acetate (0.17 g, 0.75 mmol) and 2-methyl-8-hydroxyquinoline (0.24 g, 1.5 mmol) were loaded into a convection tube; the tube was filled with dry methanol and kept at 333 K. Crystals were collected from the side arm after several days. Although well formed, all specimens had a slightly blemished interior.

### Refinement

The crystal used in the study was a multiply twinned crystal. The diffraction intensities were separated with the RLATT routine of the data collection software, and that component that diffracted to the highest 2θ limit was selected for integration. Although the specimen diffracted strongly, with a high proportion of 'observeds', there was serious overlapping between the main component and the minor components, particularly at low angles.

Carbon-bound H-atoms were placed in calculated positions (C—H 0.95 to 0.98 Å; O–H 0.84 Å) and were included in the refinement in the riding model approximation, with *U*(H) set to 1.2 to 1.5*U*(C, O).

The final difference Fourier map had a large peak/deep hole in the vicinity of the Zn1 atom. These could not be reduced even with the 2θ maximum was lowered to 50 °.

### Crystal data

**Table:**

[Zn_2_(C_10_H_8_NO)_2_(C_2_H_3_O_2_)_2_(CH_4_O)_2_]	*Z* = 1
*M**_r_* = 629.26	*F*(000) = 324
Triclinic, *P*1	*D*_x_ = 1.647 Mg m^−^^3^
Hall symbol: -P 1	Mo *K*α radiation, λ = 0.71073 Å
*a* = 6.9496 (1) Å	Cell parameters from 3551 reflections
*b* = 9.6262 (2) Å	θ = 2.2–28.3°
*c* = 9.8232 (2) Å	µ = 1.95 mm^−^^1^
α = 75.241 (1)°	*T* = 100 K
β = 89.688 (1)°	Block, yellow
γ = 86.596 (1)°	0.38 × 0.28 × 0.18 mm
*V* = 634.32 (2) Å^3^	

### Data collection

**Table:**

Bruker SMART APEX diffractometer	2855 independent reflections
Radiation source: fine-focus sealed tube	2534 reflections with *I* > 2σ(*I*)
graphite	*R*_int_ = 0.042
ω scans	θ_max_ = 27.5°, θ_min_ = 2.1°
Absorption correction: multi-scan (*SADABS*; Sheldrick, 1996)	*h* = −9→9
*T*_min_ = 0.525, *T*_max_ = 0.721	*k* = −12→12
5601 measured reflections	*l* = −12→12

### Refinement

**Table:**

Refinement on *F*^2^	Primary atom site location: structure-invariant direct methods
Least-squares matrix: full	Secondary atom site location: difference Fourier map
*R*[*F*^2^ > 2σ(*F*^2^)] = 0.076	Hydrogen site location: inferred from neighbouring sites
*wR*(*F*^2^) = 0.230	H-atom parameters constrained
*S* = 1.13	*w* = 1/[σ^2^(*F*_o_^2^) + (0.1574*P*)^2^ + 1.7954*P*] where *P* = (*F*_o_^2^ + 2*F*_c_^2^)/3
2855 reflections	(Δ/σ)_max_ = 0.001
175 parameters	Δρ_max_ = 3.72 e Å^−^^3^
0 restraints	Δρ_min_ = −1.85 e Å^−^^3^

### Fractional atomic coordinates and isotropic or equivalent isotropic displacement parameters (Å^2^)

**Table:**

	*x*	*y*	*z*	*U*_iso_*/*U*_eq_	
Zn1	0.57254 (8)	0.63131 (6)	0.87572 (5)	0.0148 (3)	
O1	0.5349 (5)	0.4205 (4)	0.9193 (4)	0.0174 (8)	
O2	0.4331 (6)	0.8211 (4)	0.8160 (4)	0.0201 (8)	
O3	0.1619 (6)	0.7137 (5)	0.8017 (4)	0.0236 (9)	
O4	0.8356 (6)	0.6830 (5)	0.9340 (4)	0.0233 (9)	
H4	0.9089	0.7231	0.8695	0.028*	
N1	0.6717 (6)	0.5859 (5)	0.6847 (5)	0.0165 (9)	
C1	0.5872 (7)	0.3551 (6)	0.8200 (5)	0.0169 (10)	
C2	0.5764 (8)	0.2097 (6)	0.8320 (6)	0.0184 (10)	
H2	0.5313	0.1493	0.9168	0.022*	
C3	0.6315 (9)	0.1497 (6)	0.7196 (6)	0.0229 (11)	
H3	0.6238	0.0492	0.7303	0.028*	
C4	0.6955 (8)	0.2334 (6)	0.5956 (6)	0.0232 (11)	
H4A	0.7301	0.1912	0.5207	0.028*	
C5	0.7104 (8)	0.3827 (6)	0.5786 (6)	0.0191 (10)	
C6	0.6596 (7)	0.4431 (6)	0.6917 (5)	0.0150 (9)	
C7	0.7707 (8)	0.4776 (6)	0.4547 (6)	0.0196 (11)	
H7	0.8029	0.4427	0.3748	0.023*	
C8	0.7834 (8)	0.6205 (6)	0.4485 (5)	0.0208 (11)	
H8	0.8248	0.6846	0.3646	0.025*	
C9	0.7341 (8)	0.6730 (6)	0.5687 (5)	0.0178 (10)	
C10	0.7542 (9)	0.8281 (6)	0.5643 (6)	0.0227 (11)	
H10A	0.7336	0.8427	0.6586	0.034*	
H10B	0.6582	0.8879	0.4988	0.034*	
H10C	0.8838	0.8552	0.5326	0.034*	
C11	0.2496 (8)	0.8207 (6)	0.8104 (5)	0.0179 (10)	
C12	0.1384 (9)	0.9585 (7)	0.8161 (7)	0.0297 (13)	
H12A	0.2244	1.0381	0.7939	0.045*	
H12B	0.0864	0.9488	0.9108	0.045*	
H12C	0.0322	0.9780	0.7474	0.045*	
C13	0.9045 (8)	0.6565 (7)	1.0750 (6)	0.0233 (11)	
H13A	1.0426	0.6285	1.0786	0.035*	
H13B	0.8350	0.5787	1.1356	0.035*	
H13C	0.8832	0.7441	1.1079	0.035*	

### Atomic displacement parameters (Å^2^)

**Table:**

	*U*^11^	*U*^22^	*U*^33^	*U*^12^	*U*^13^	*U*^23^
Zn1	0.0190 (4)	0.0192 (4)	0.0083 (4)	−0.0030 (2)	0.0016 (2)	−0.0068 (2)
O1	0.027 (2)	0.0212 (18)	0.0063 (16)	−0.0035 (15)	0.0078 (14)	−0.0071 (14)
O2	0.025 (2)	0.0206 (19)	0.0146 (17)	−0.0033 (15)	0.0021 (15)	−0.0033 (14)
O3	0.023 (2)	0.032 (2)	0.020 (2)	−0.0040 (16)	0.0045 (16)	−0.0146 (17)
O4	0.0186 (19)	0.041 (2)	0.0123 (18)	−0.0090 (16)	0.0026 (14)	−0.0094 (16)
N1	0.018 (2)	0.024 (2)	0.0098 (19)	−0.0017 (16)	−0.0004 (15)	−0.0079 (16)
C1	0.018 (2)	0.024 (3)	0.012 (2)	−0.0030 (19)	0.0016 (18)	−0.0084 (19)
C2	0.023 (3)	0.019 (2)	0.015 (2)	−0.0026 (19)	0.0028 (19)	−0.0058 (19)
C3	0.030 (3)	0.021 (3)	0.021 (3)	0.001 (2)	−0.002 (2)	−0.012 (2)
C4	0.025 (3)	0.029 (3)	0.020 (3)	−0.001 (2)	0.000 (2)	−0.015 (2)
C5	0.017 (2)	0.027 (3)	0.016 (2)	0.000 (2)	−0.0011 (19)	−0.012 (2)
C6	0.017 (2)	0.021 (2)	0.008 (2)	0.0006 (18)	0.0004 (17)	−0.0055 (18)
C7	0.018 (2)	0.032 (3)	0.012 (2)	0.002 (2)	0.0005 (19)	−0.012 (2)
C8	0.020 (3)	0.033 (3)	0.010 (2)	−0.001 (2)	−0.0003 (19)	−0.008 (2)
C9	0.018 (2)	0.027 (3)	0.009 (2)	−0.003 (2)	0.0009 (18)	−0.0058 (19)
C10	0.035 (3)	0.023 (3)	0.012 (2)	−0.006 (2)	0.003 (2)	−0.006 (2)
C11	0.020 (2)	0.024 (3)	0.010 (2)	0.0004 (19)	0.0012 (18)	−0.0057 (19)
C12	0.027 (3)	0.027 (3)	0.034 (3)	0.002 (2)	0.002 (2)	−0.006 (2)
C13	0.023 (3)	0.035 (3)	0.013 (2)	−0.004 (2)	0.001 (2)	−0.009 (2)

### Geometric parameters (Å, °)

**Table:**

Zn1—O1	1.997 (4)	C4—C5	1.414 (8)
Zn1—O2	1.968 (4)	C4—H4A	0.9500
Zn1—O1^i^	2.092 (3)	C5—C7	1.402 (8)
Zn1—O4	2.045 (4)	C5—C6	1.413 (7)
Zn1—N1	2.134 (4)	C7—C8	1.369 (8)
O1—C1	1.328 (6)	C7—H7	0.9500
O1—Zn1^i^	2.092 (3)	C8—C9	1.431 (7)
O2—C11	1.277 (7)	C8—H8	0.9500
O3—C11	1.250 (7)	C9—C10	1.497 (7)
O4—C13	1.423 (6)	C10—H10A	0.9800
O4—H4	0.8400	C10—H10B	0.9800
N1—C9	1.319 (7)	C10—H10C	0.9800
N1—C6	1.366 (7)	C11—C12	1.508 (8)
C1—C2	1.381 (7)	C12—H12A	0.9800
C1—C6	1.435 (7)	C12—H12B	0.9800
C2—C3	1.412 (7)	C12—H12C	0.9800
C2—H2	0.9500	C13—H13A	0.9800
C3—C4	1.366 (9)	C13—H13B	0.9800
C3—H3	0.9500	C13—H13C	0.9800
			
O1—Zn1—O1^i^	75.2 (2)	C6—C5—C4	119.1 (5)
O1—Zn1—O2	142.5 (2)	N1—C6—C5	122.8 (5)
O1—Zn1—O4	114.7 (2)	N1—C6—C1	116.8 (4)
O1—Zn1—N1	79.8 (2)	C5—C6—C1	120.4 (5)
O1^i^—Zn1—O2	95.8 (2)	C8—C7—C5	120.2 (5)
O1^i^—Zn1—O4	94.5 (2)	C8—C7—H7	119.9
O1^i^—Zn1—N1	155.0 (2)	C5—C7—H7	119.9
O2—Zn1—O4	102.1 (2)	C7—C8—C9	119.9 (5)
O2—Zn1—N1	104.7 (2)	C7—C8—H8	120.1
O4—Zn1—N1	95.0 (2)	C9—C8—H8	120.1
C1—O1—Zn1	116.2 (3)	N1—C9—C8	120.7 (5)
C1—O1—Zn1^i^	139.0 (3)	N1—C9—C10	119.2 (5)
Zn1—O1—Zn1^i^	104.81 (16)	C8—C9—C10	120.1 (5)
C11—O2—Zn1	116.0 (3)	C9—C10—H10A	109.5
C13—O4—Zn1	125.3 (3)	C9—C10—H10B	109.5
C13—O4—H4	117.3	H10A—C10—H10B	109.5
Zn1—O4—H4	117.3	C9—C10—H10C	109.5
C9—N1—C6	119.7 (4)	H10A—C10—H10C	109.5
C9—N1—Zn1	130.0 (4)	H10B—C10—H10C	109.5
C6—N1—Zn1	110.3 (3)	O3—C11—O2	123.5 (5)
O1—C1—C2	124.6 (5)	O3—C11—C12	120.0 (5)
O1—C1—C6	117.0 (5)	O2—C11—C12	116.5 (5)
C2—C1—C6	118.4 (5)	C11—C12—H12A	109.5
C1—C2—C3	120.8 (5)	C11—C12—H12B	109.5
C1—C2—H2	119.6	H12A—C12—H12B	109.5
C3—C2—H2	119.6	C11—C12—H12C	109.5
C4—C3—C2	121.2 (5)	H12A—C12—H12C	109.5
C4—C3—H3	119.4	H12B—C12—H12C	109.5
C2—C3—H3	119.4	O4—C13—H13A	109.5
C3—C4—C5	120.1 (5)	O4—C13—H13B	109.5
C3—C4—H4A	119.9	H13A—C13—H13B	109.5
C5—C4—H4A	119.9	O4—C13—H13C	109.5
C7—C5—C6	116.7 (5)	H13A—C13—H13C	109.5
C7—C5—C4	124.2 (5)	H13B—C13—H13C	109.5
			
O2—Zn1—O1—C1	102.0 (4)	C6—C1—C2—C3	−1.3 (8)
O4—Zn1—O1—C1	−89.7 (4)	C1—C2—C3—C4	−0.5 (9)
O1^i^—Zn1—O1—C1	−177.9 (5)	C2—C3—C4—C5	0.9 (9)
N1—Zn1—O1—C1	1.1 (4)	C3—C4—C5—C7	−178.6 (6)
O2—Zn1—O1—Zn1^i^	−80.1 (3)	C3—C4—C5—C6	0.5 (8)
O4—Zn1—O1—Zn1^i^	88.17 (19)	C9—N1—C6—C5	−0.4 (8)
O1^i^—Zn1—O1—Zn1^i^	0.0	Zn1—N1—C6—C5	178.2 (4)
N1—Zn1—O1—Zn1^i^	179.0 (2)	C9—N1—C6—C1	−178.9 (5)
O1—Zn1—O2—C11	7.8 (5)	Zn1—N1—C6—C1	−0.3 (6)
O4—Zn1—O2—C11	−161.4 (4)	C7—C5—C6—N1	−1.5 (8)
O1^i^—Zn1—O2—C11	−65.4 (4)	C4—C5—C6—N1	179.3 (5)
N1—Zn1—O2—C11	100.1 (4)	C7—C5—C6—C1	176.9 (5)
O2—Zn1—O4—C13	107.2 (4)	C4—C5—C6—C1	−2.3 (8)
O1—Zn1—O4—C13	−65.5 (5)	O1—C1—C6—N1	1.2 (7)
O1^i^—Zn1—O4—C13	10.3 (4)	C2—C1—C6—N1	−178.8 (5)
N1—Zn1—O4—C13	−146.6 (4)	O1—C1—C6—C5	−177.3 (5)
O2—Zn1—N1—C9	36.2 (5)	C2—C1—C6—C5	2.7 (8)
O1—Zn1—N1—C9	178.0 (5)	C6—C5—C7—C8	1.8 (8)
O4—Zn1—N1—C9	−67.7 (5)	C4—C5—C7—C8	−179.1 (5)
O1^i^—Zn1—N1—C9	−179.6 (4)	C5—C7—C8—C9	−0.3 (8)
O2—Zn1—N1—C6	−142.2 (3)	C6—N1—C9—C8	2.1 (8)
O1—Zn1—N1—C6	−0.4 (3)	Zn1—N1—C9—C8	−176.2 (4)
O4—Zn1—N1—C6	113.9 (3)	C6—N1—C9—C10	−177.6 (5)
O1^i^—Zn1—N1—C6	1.9 (6)	Zn1—N1—C9—C10	4.1 (8)
Zn1—O1—C1—C2	178.4 (4)	C7—C8—C9—N1	−1.8 (8)
Zn1^i^—O1—C1—C2	1.5 (9)	C7—C8—C9—C10	177.9 (5)
Zn1—O1—C1—C6	−1.6 (6)	Zn1—O2—C11—O3	−20.5 (7)
Zn1^i^—O1—C1—C6	−178.5 (4)	Zn1—O2—C11—C12	159.0 (4)
O1—C1—C2—C3	178.7 (5)		

Symmetry codes: (i) −*x*+1, −*y*+1, −*z*+2.

### Hydrogen-bond geometry (Å, °)

**Table:**

*D*—H···*A*	*D*—H	H···*A*	*D*···*A*	*D*—H···*A*
O4—H4···O3^ii^	0.84	1.88	2.602 (6)	143

Symmetry codes: (ii) *x*+1, *y*, *z*.
